# Synthesis of Tosyl- and Nosyl-Ended Polyisobutylenes with High Extent of Functionalities: The Effect of Reaction Conditions

**DOI:** 10.3390/polym12112504

**Published:** 2020-10-28

**Authors:** Balázs Pásztói, Tobias M. Trötschler, Ákos Szabó, Györgyi Szarka, Benjamin Kerscher, Rolf Mülhaupt, Béla Iván

**Affiliations:** 1 Polymer Chemistry Research Group, Institute of Materials and Environment Chemistry, Research Centre for Natural Sciences, Magyar tudósok körútja 2, H-1117 Budapest, Hungary; szabo.akos@ttk.hu (Á.S.); szarka.gyorgyi@ttk.hu (G.S.); 2 George Hevesy PhD School of Chemistry, Institute of Chemistry, Faculty of Science, Eötvös Loránd University, Pázmány Péter sétány 2, H-1117 Budapest, Hungary; 3 Institute for Macromolecular Chemistry, University of Freiburg, Stefan-Meier-Str. 31, D-79104 Freiburg, Germany; ttroet@yahoo.de (T.M.T.); benjamin_kerscher@web.de (B.K.); 4 Freiburg Materials Research Center, University of Freiburg, Stefan-Meier-Str. 21, D-79104 Freiburg, Germany; 5 Freiburg Center for Interactive Materials and Bioinspired Technologies (FIT), University of Freiburg, Georges-Köhler-Allee 105, D-79110 Freiburg, Germany

**Keywords:** isobutylene, tosyl-ended polyisobutylene, nosyl-ended polyisobutylene, quasiliving carbocationic polymerization (QLCCP), allyl-terminated polyisobutylene, exo-olefin ended polyisobutylene, hydroxyl-ended polyisobutylene, 4-dimethylaminopyridine (DMAP), trimethylamine (TEA)

## Abstract

Endfunctional polymers possess significant industrial and scientific importance. Sulfonyl endgroups, such as tosyl and nosyl endfunctionalities, due their ease of substitution are highly desired for a variety of polymer structures. The sulfonylation of hydroxyl-terminated polyisobutylene (PIB-OH), a chemically and thermally stable, biocompatible, fully saturated polymer, with tosyl chloride (TsCl) and nosyl chloride (NsCl) is presented in this study. PIB-OHs derived from commercial exo-olefin-ended PIB (PIB_exo_-OH) and allyl-terminated polymer made via quasiliving carbocationic polymerization of isobutylene (PIB_all_-OH) were tosylated and nosylated in the presence of 4-dimethylaminopyridine (DMAP), pyridine and 1-methylimidazole (1-MI) catalysts and triethylamine (TEA). Our systematic investigations revealed that the end product distribution strongly depends on the relative amount of the components, especially that of TEA. While PIB_exo_-OTs with quantitative endfunctionality is readily formed from PIB_exo_-OH, its nosylation is not as straightforward. During sulfonylation of PIB_all_-OH, the formed tosyl and nosyl endgroups are easily substituted with chloride ions, formed in the first step of sulfonylation, leading to chloride termini. We found that decreased amounts of TEA afford the synthesis of PIB_all_-OTs and PIB_all_-ONs with higher than 90% endfunctionalities. These sulfonyl-ended PIBs open new ways for utilizing PIB in various fields and in the synthesis of novel PIB-containing macromolecular architectures.

## 1. Introduction

Functional polymers with terminal and or pendant functionalities have significant industrial and scientific importance, and as a consequence, intensive research and developments have been taking place with such polymers worldwide. Among these macromolecular materials, functional polyisobutylenes (PIBs) have gained remarkable interest in the last couple of years (see e.g., Refs. 1-30 and references therein). This is mainly due to the demands to increase the average endfunctionality by either conventional or quasiliving carbocationic polymerizations, or to the utilization of functional PIBs as building blocks in a variety of new materials with advanced application possibilities. PIB has a fully saturated, chemically inert backbone with low glass transition temperature, high thermal and oxidative stability, outstanding barrier properties, etc. Based on these attractive features, functional PIBs and its copolymers have already gained broad fields of applications, e.g., starting material of butyl rubbers, oil, fuel and lubricant additives, sealants, adhesives, insulating materials, and the component of biomedical devices on the basis of its biocompatibility.

**Scheme 1:**
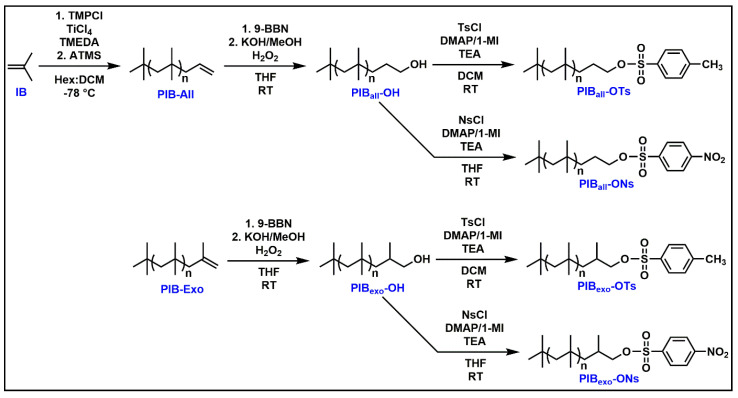
The synthesis routes for the preparation of tosyl- and nosyl-ended polyisobutylenes derived from exo-olefin-ended (PIB-Exo) and allyl-terminated (PIB-All) polymers (PIB-All was obtained by quasiliving carbocationic polymerization).

In spite of the availability of PIBs with a wide range of endfunctionalities [1,2,3,4,5,6,7,8,9,10,11,12,13,14,15,16,17,18,19,20,21,22,23,24,25,26,27,28,29,30,31,32,33,34,35,36], a reliable process for certain highly desired endfunctional PIBs with high extents of reactive chain end functionalities, for instance, organosulfonates, such as tosyl- or nosyl-ended PIBs, are still lacking. The most convenient way to obtain PIB-sulfonates is the sulfonylation of hydroxyl-terminated PIBs (PIB-OHs) by the corresponding sulfonyl chlorides, e.g., tosyl chloride (TsCl) or nosyl chloride (NsCl) as shown in Scheme 1. PIB-OHs are usually prepared by hydroboration/oxidation of olefin-terminated PIBs [31,32]. While commercial PIBs with relatively broad molecular weight distributions (MWD) have about 80% reactive exo-olefin functionality [1,4,5,6,8,9] (PIB-Exo), the inifer method [33] and quasiliving carbocationic polymerization (QLCCP) results in PIB-Exo with quantitative vinylidene endgroups [34,35]. In situ allylation by endquenching of QLCCP of isobutylene with allyltrimethylsilane yields PIBs directly with allyl termini (PIB-All) [32]. It has to be noted that significant efforts have been made to increase the exo-olefin functionality in PIB-Exo by conventional carbocationic polymerization in recent years [1,4,5,6,9,24,25,26,27,28,29]. Both PIB-Exo and PIB-All were converted to PIB-OHs [31,32,36], which in principle can be converted to PIB-sulfonates, e.g., tosylates, nosylates and mesylates. The interest in such PIBs is based on the fact that alkyl tosylates are among the most versatile compounds for substitution reactions because the tosyl group is an excellent leaving group. As widely accepted, tosylates can be prepared by the reaction of tosyl chloride and an alcohol. Typically, bases promote this process by capturing the generated HCl during the reaction. Catalysts, such as 4-dialkylaminopyridines, like 4-dimethylaminopyridine (DMAP) and tertiary amines, e.g., triethylamine (TEA), proved to be effective for a wide range of species [37,38,39]. Furthermore, imidazole-based sulfonylation was also described [40,41]. Although these catalysts are efficient in tosylation of alcohols of low molecular weights in most cases, undesired side reactions were observed for some species and under certain reaction conditions. Mostly, the formed tosyl group is substituted by the chloride anion yielding chloride functional group [39,42]. While tosylates are preferred in organic reactions and have a quite extensive literature background, nosylates, despite their even better synthetic features in substitution reactions [43,44,45], have not been explored intensively so far. Based on these characteristics of tosylates and nosylates, effective tosylation/nosylation of PIB-OHs is expected to lead to PIBs with tosyl and nosyl endfunctionalities. These can be utilized in a wide range of subsequent substitution reactions and thus in the preparation of a variety of new macromolecular materials. It has to be mentioned that tosylates and nosylates can also be applied as macroinitiators, e.g., for quasiliving ring-opening polymerization of oxazolines [46]. Consequently, PIBs with tosyl or nosyl chain ends would provide unique opportunities to prepare an array of novel macromolecular materials.

Based on the current literature, very limited knowledge exists on PIBs with sulfonate (tosylate, nosylate, mesylate) endgroups. In an early attempt, linear and three-arm star PIB-OHs were tosylated with excess TsCl in the presence of DMAP and TEA as bases in dichloromethane (DCM) [47]. Although complete consumption of the hydroxyl groups was claimed, detailed analysis of the products was not carried out. However, it was found that using these tosylated PIBs as macroinitiators for the quasiliving cationic ring-opening polymerization (CROP) of 2-methyl-2-oxazoline resulted only in 70–80% blocking efficiency, indicating incomplete initiation of the CROP process by the chain ends, which might be an indication of incomplete tosylation. As found by us recently [48] and during our preliminary experiments, lower than quantitative endfunctionalization was achieved by reacting TsCl with PIB-OH under similar conditions as reported [47]. Converting the hydroxyl group of PIB-OH prepared from industrial PIB-Exo by methanesulfonyl chloride (mesyl chloride, MsCl) led to mesylated PIB, which was used as an intermediate for polyisobutylene supported catalyst systems [49,50]. However, detailed analysis of this process and the resulting polymers or the average endfunctionality has not been reported in the case of these sulfonylations.

Herein, we report on our systematic investigations aiming at to determine the effect of reaction conditions on the efficiency of tosylation and nosylation of PIB-OHs derived from commercially available PIB-Exo and laboratory PIB-All prepared by QLCCP. Our definite goal was to reveal the reaction parameters which result in high tosylation and nosylation yields, i.e., high tosyl and nosyl endfunctionalities. Thorough experiments were also carried out on the influence of the origin of the PIB-OH, i.e., PIB_exo_-OH or PIB_all_-OH, the ratio of the reagents and the reaction times on the endfunctionalities of the sulfonylated PIBs.

## 2. Materials and Methods

### 2.1. Materials

Dichloromethane (DCM, 99.9%, Molar Chemicals, Halásztelek, Hungary), tetrahydrofuran (THF, 99.9%, VWR Chemicals, West Chester, PA, USA), benzotrifluoride (BTF, ≥99%, Sigma-Aldrich, St. Louis, MO, USA) and 2-ethyl-2-oxazoline (EtOx, 99+%, Aldrich, St. Louis, MO, USA) were refluxed on CaH_2_ for a couple of hours and distilled over it under N_2_ atmosphere freshly before use. Triethylamine (TEA, ≥99.0%, TCI Chemicals, Tokyo, Japan) and 1-methylimidazole (1-MI, ≥99%, Sigma-Aldrich, St. Louis, MO, USA) was distilled under N_2_ atmosphere freshly before use. Glissopal 1000 (BASF SE, Ludwigshafen, Germany), p-toluenesulfonyl chloride (tosyl chloride, TsCl, ≥99.0%, TCI Chemicals, Tokyo, Japan), p-nitrobenzenesulfonyl chloride (nosyl chloride, NsCl, 97%, Sigma-Aldrich, St. Louis, MO, USA) and 4-dimethylaminopyridine (DMAP, ≥99.9%, Sigma-Aldrich, St. Louis, MO, USA) were used as receive.

### 2.2. Characterization

NMR spectroscopy. ^1^H NMR spectra of all endfunctional PIBs were recorded on a Varian 500 MHz spectrometer. All measurements were performed in CDCl_3_ as solvent and at 30 °C. For spectra calibration of the ^1^H NMR spectra, the chloroform peak was set to 7.26 ppm.

Gelpermeation chromatography (GPC). The GPC equipment was composed of a Waters 515 HPLC pump, Waters Styragel column set with three columns (HR1, HR2, HR4), and it was equipped with an Aligent 390 RI detector. THF was used as mobile phase with a flow rate of 1 mL/min. The average molar masses and the polydispersity (M_w_/M_n_), were determined by the use of a calibration made with narrow MWD polystyrene standards in the molecular weight range of 104 to 6 × 10^5^ Da.

### 2.3. Synthesis of PIB-All

A solution of 2-chloro-2,4,4-trimethylpentane (TMPCl, 4.1 g, 0.03 mol) in n-hexane (1520 mL) and DCM (1240 mL) was cooled down in a dry ice-isopropanol mixture to −78 °C. To this reaction solution, under continuous stirring, tetramethylethylenediamine (TMEDA, 4.1 mL, 3.2 g, 0.03 mol, 1.0 eq.) was given. Afterward, TiCl_4_ (18.2 mL, 31.4 g, 0.17 mol, 6.0 eq.) and isobutylene (IB, 32.6 mL, 23.5 g, 0.42 mol, 15.2 eq.) were carefully added and the mixture was stirred at −78 °C for 30 min. After complete conversion of IB, pre-chilled allyltrimethylsilane (ATMS, 8.8 mL, 8.3 g, 0.06 mol, 2 eq.) was given and the solution was stirred for 30 min. Finally, the reaction was stopped with the addition of cold MeOH (200 mL). The mixture was warmed up to room temperature, which resulted a two-phase mixture (including a higher phase of n-hexane and a lower phase of DCM/MeOH). The n-hexane phase was separated and washed with a solution of NaHCO_3_ in H_2_O (250 mL) three times, dried over MgSO_4_ overnight and cleaned up via filtering. The solvent was removed under reduced pressure and the product was dried under vacuum at 60 °C until constant weight. PIB-All was obtained as a colourless, clean, viscous liquid (yield: 22.4 g, 81%). ^1^H NMR (500 MHz, CDCl_3_, 30 °C): δ = 0.76–1.80 (m, 116H), 1.95–2.07 (d, 2H), 4.92-5.07 (m, 2H), 5.77-5.93 (m, 1H) ppm. GPC: M_n_ = 820 g/mol, D = 1.16).

### 2.4. Synthesis of PIB_all_-OH

PIB-All (7.0 g, 7.78 mmol) was dissolved in dry THF (35 mL) under nitrogen atmosphere. Afterwards, 0.5 M solution of 9-borabicyclo[3.3.1]nonane in THF (9-BBN, 0.5 M, 78 mL, 0.039 mol, 5 eq.) was added dropwise and the mixture was stirred for 5 h at room temperature. Into this mixture, KOH (6.5 g, 0.12 mol, 15 eq.) in MeOH solution (43 mL) was carefully added, then the reaction mixture was cooled down with an ice bath, and aqueous H_2_O_2_ (30%, 13.2 mL, 4.0 g, 0.12 mol, 15 eq.) was dropped under constant stirring. The reaction mixture was stirred overnight at room temperature. After this, n-hexane (70 mL) and H_2_O (20 mL) were added, and the organic phase of the two-phase mixture was separated, washed with a solution of NaHCO_3_ in H_2_O (20 mL) three times, dried over MgSO_4_ overnight and cleaned up via filtering. The solvent was removed under reduced pressure and the product was dried under vacuum at 60 °C until constant weight. PIB_all_-OH was obtained as a colourless, clean, viscous liquid (yield: 6.9 g, 99%). ^1^H NMR (500 MHz, CDCl_3_, 30 °C): δ = 0.78–1.91 (m, 130H), 3.55–3.68 (t, 2H). GPC: M_n_ = 1160 g/mol, D = 1.18.

### 2.5. Synthesis of PIB_exo_-OH

PIB-Exo (50.0 g, 50.0 mmol) was dissolved in dry THF (250 mL) under nitrogen atmosphere. Afterward, 0.5 M solution of 9-borabicyclo[3.3.1]nonane in THF (9-BBN, 0.5 M, 500 mL, 0.250 mol, 5 eq.) was added dropwise and the mixture was stirred for 4 h at room temperature. Into this mixture, KOH (70.1 g, 1.25 mol, 25 eq.) in MeOH solution (440 mL) was carefully added, then the reaction mixture was cooled down with an ice bath, and aqueous H_2_O_2_ (30%, 29.3 mL, 42.5 g, 1.25 mol, 25 eq.) was dropped under constant stirring. The reaction mixture was stirred overnight at room temperature. After this, n-hexane (250 mL) and H_2_O (100 mL) were added, and the organic phase of the two-phase mixture was separated, washed with a solution of NaHCO_3_ in H_2_O (100 mL) three times, dried over MgSO_4_ overnight and cleaned up via filtering. The solvent was removed under reduced pressure and the product was dried under vacuum at 60 °C until constant weight. The final product was resulted after precipitation from THF to tenfold excess of MeOH. PIB_exo_-OH was obtained as a colorless, clean, viscous liquid (yield: 43.6 g, 86%). ^1^H NMR (500 MHz, CDCl_3_, 30 °C): δ = 0.88–1.80 (m, 216H), 3.26–3.53 (m, 2H), 4.77–4.86 (*coupled, m, 1H), 5.09–5.19 (*endo, m, 1H). GPC: M_n_ = 1360 g/mol, D = 1.53.

### 2.6. Experiments on the Tosylation of PIB_exo_-OH and PIB_all_-OH

0.1 g or 0.2 g of PIB-OH was dissolved in dry DCM and the solution was added to a previously heat-dried small glass vial equipped with a magnetic stirrer. The vial was carefully closed with a septum cap and nitrogen was transferred through solution for several minutes. Afterwards, calculated amount of TEA and the catalyst (DMAP or 1-methylimidazole in DCM solution) were added with constant stirring. Finally, tosyl chloride in DCM solution was given dropwise to the reaction mixture. Reaction was conducted at room temperature; samples were withdrawn at defined times and precipitated into large excess of MeOH. Polymer was isolated, washed with MeOH and dried under vacuum at 40 °C until constant weight. Dry product was analyzed by ^1^H NMR spectroscopy to determine the conversion of hydroxyl endgroup.

### 2.7. Experiments on the Nosylation of PIB_exo_-OH and PIB_all_-OH

0.1 g or 0.2 g of PIB-OH was dissolved in dry THF and the solution was added to a previously heat-dried small glass vial equipped with a magnetic stirrer. The vial was carefully closed with a septum cap, and nitrogen was transferred through solution for several minutes. Afterward, calculated amount of TEA and the catalyst (DMAP or 1-methylimidazole in THF solution) were added with constant stirring. Finally, nosyl chloride in THF solution was given dropwise to the reaction mixture. Reaction was conducted at room temperature; samples were withdrawn at defined times and precipitated into large excess of MeOH. Polymer was isolated, washed with MeOH and dried under vacuum at 40 °C until constant weight. The dry product was analyzed by ^1^H NMR spectroscopy to determine the conversion of the hydroxyl endgroup.

### 2.8. Synthesis of PIB_all_-OTs

PIB_all_-OH (37.0 g, 30.8 mmol) was dissolved in dry DCM (370 mL) under nitrogen atmosphere. Afterwards, TEA (21.5 mL, 15.6 g, 0.15 mol, 5 eq.) and 1-methylimidazole (4.9 mL, 5.1 g, 62 mmol, 2 eq.) were added under continuous stirring. After complete dissolution of the reagents, tosyl chloride (58.8 g, 0.31 mol, 10 eq.) in DCM solution was dropped into the reaction mixture carefully while cooled down with an ice-bath. The reaction content was warmed up to room temperature and stirred overnight. After the addition of MeOH (50 mL), the mixture was stirred for 30 min, concentrated and precipitated into tenfold excess of MeOH (2000 mL). Raw product was isolated, washed with MeOH (100 mL) several times, dried under vacuum at 60 °C. Then it was resolved in n-hexane (150 mL), filtered if necessary, precipitated again into tenfold excess of MeOH (1500 mL), the product was isolated, washed with MeOH (100 mL) several times and dried under vacuum at 60 °C until constant weight. Precipitation procedure was usually conducted three times, but until the dry product was completely clear and lost the initial yellowish colour. PIB_all_-OTs was obtained as a colourless, clean, viscous liquid (yield: 36.7 g, 88%). ^1^H NMR (500 MHz, CDCl_3_, 30 °C): δ = 0.65–1.68 (m, 192H), 2.39–2.50 (s, 3H), 3.92–4.07 (t, 2H), 7.31–7.38 (d, 2H), 7.73–7.87 (d, 2H) ppm. GPC: M_n_ = 1480 g/mol, D = 1.16.

### 2.9. Synthesis of PIB_all_-ONs

PIB_all_-OH (3.45 g, 3.45 mmol) was dissolved in dry THF (50 mL) under nitrogen atmosphere. Afterwards, TEA (2.4 mL, 1.7 g, 0.017 mol, 5 eq.) and 1-methylimidazole (0.55 mL, 0.67 g, 6.9 mmol, 2 eq.) were added under continuous stirring. After complete dissolution of the reagents, nosyl chloride (7.65 g, 0.035 mol, 10 eq.) in THF solution was dropped into the reaction mixture carefully while cooled down with an ice-bath. The reaction content was warmed up to room temperature and stirred overnight. After the addition of MeOH (10 mL), the mixture was stirred for 30 min, concentrated and precipitated into tenfold excess of MeOH (500 mL). Raw product was isolated, washed with MeOH (20 mL) several times, dried under vacuum at 60 °C. Then it was resolved in n-hexane (35 mL), filtered if necessary, precipitated again into tenfold excess of MeOH (350 mL), the product was isolated, washed with MeOH (20 mL) several times and dried under vacuum at 60 °C until constant weight. The precipitation procedure was usually conducted three times until the dry product was completely clear. PIB_all_-ONs was obtained as a slightly yellow, clean, viscous liquid (yield: 1.55 g, 45%). ^1^H NMR (500 MHz, CDCl_3_, 30 °C): δ = 0.78–1.86 (m, 147H), 3.46–3.53 (*PIB-Cl, t, 2H), 4.07–4.16 (t, 2H), 8.07–8.18 (d, 2H), 8.36–8.45 (d, 2H) ppm. GPC: M_n_ = 1310 g/mol, D = 1.11.

## 3. Results and Discussion

**Figure 1:**
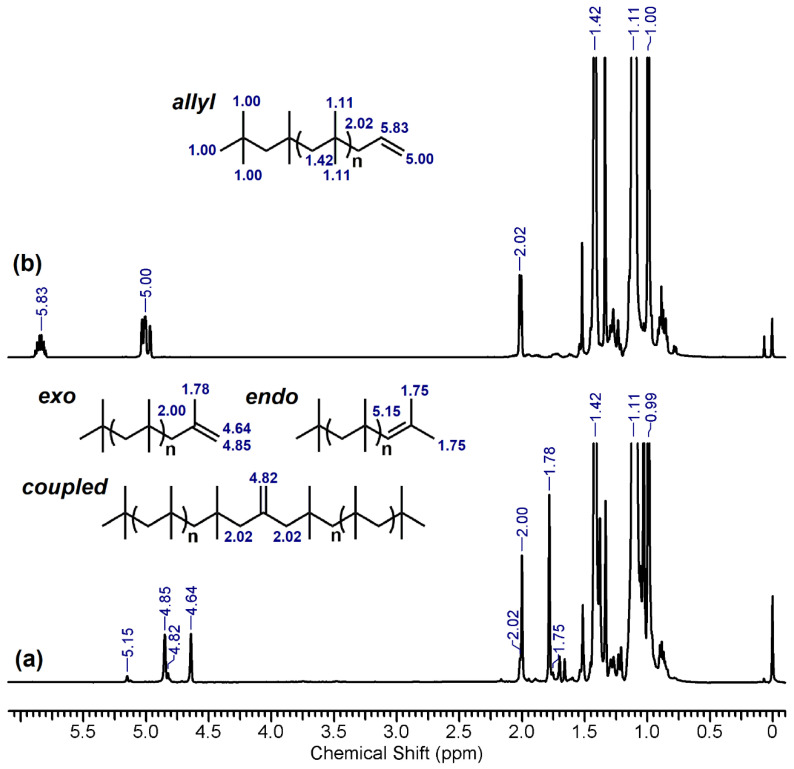
^1^H NMR spectra of the exo-olefin-ended (**a**) and allyl-terminated polyisobutylene (**b**).

**Figure 2:**
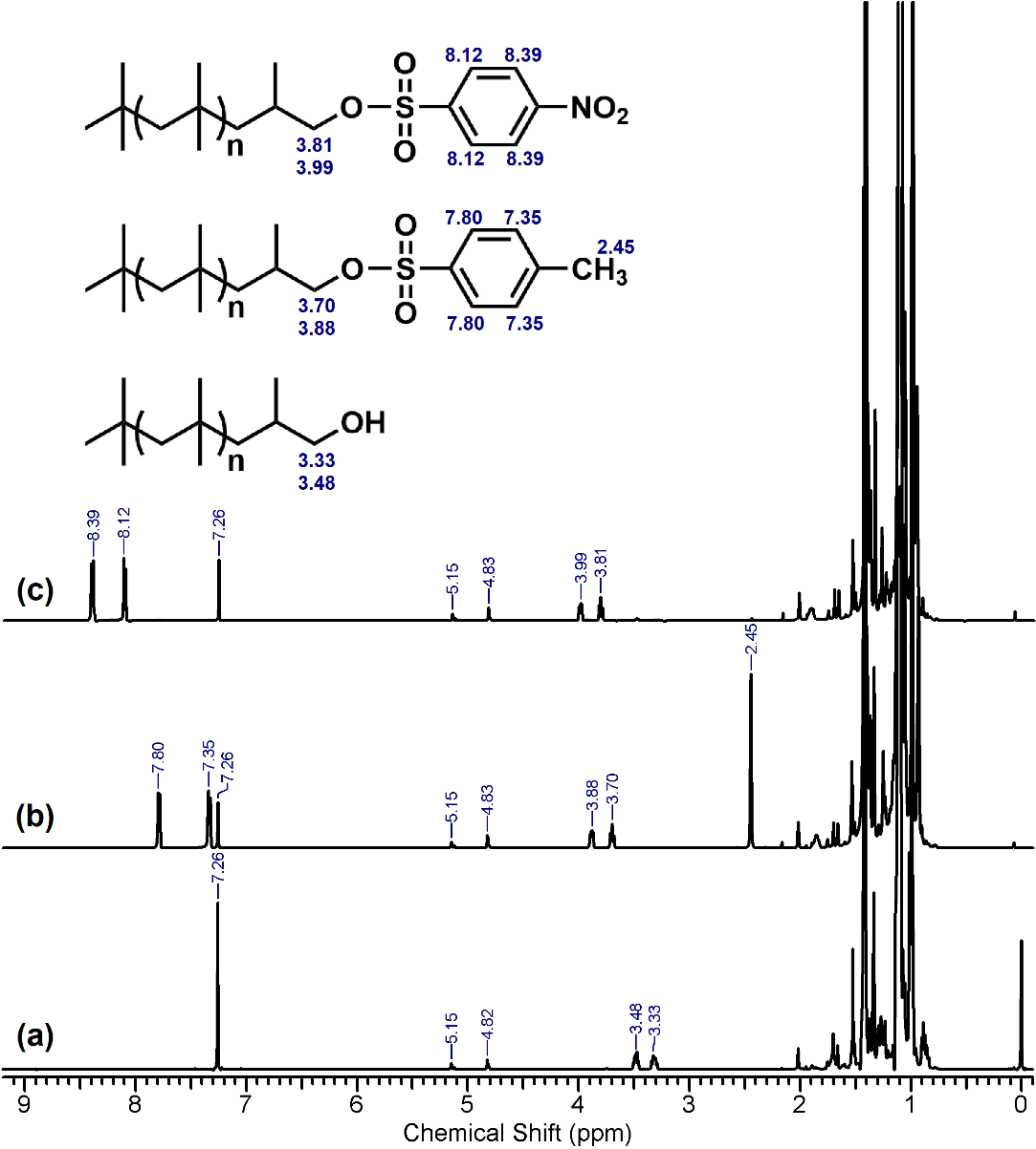
^1^H NMR spectra of PIB_exo_-OH (**a**), PIB_exo_-OTs (**b**) and PIB_exo_-ONs (**c**).

**Figure 3:**
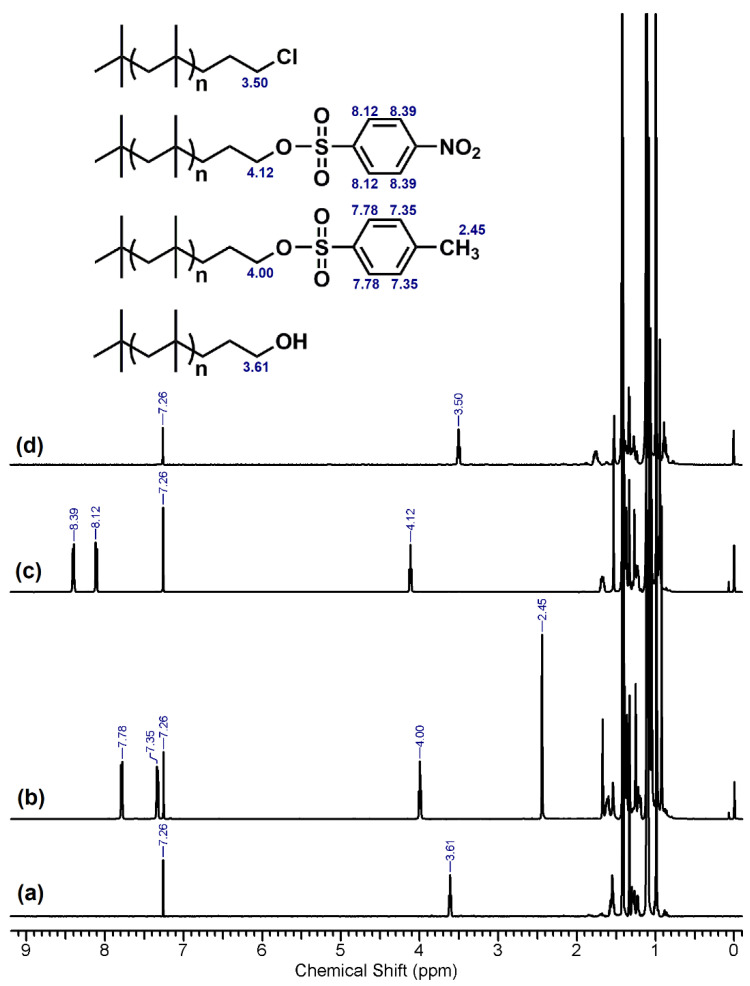
^1^H NMR spectra of PIB_all_-OH (**a**), PIB_all_-OTs (**b**), PIB_all_-ONs (**c**) and PIB_all_-Cl (**d**).

In order to study the effect of the chain end structure on tosylation and nosylation, both exo-olefin- and allyl-ended PIBs were used as starting materials. Because the exo-olefin-teminated polymer (PIB-Exo) is a commercially available inexpensive product, and used widely for research purposes as well (see e.g., references 48–50 and references therein), we selected this polymer as one of the starting materials for our studies. As displayed in Figure 1, the commercial PIB-Exo, obtained by utilizing the chain transfer process in conventional carbocationic polymerization of isobutylene, contains not only exo-, but endo- and in-chain olefins as well (see also Figure S1 in the Supporting Information). Considering these double bonds, PIB-Exo has 81% exo-olefin functionality and M_n_ of 1200 g/mol by ^1^H NMR spectroscopy (M_n_ = 1150 g/mol and M_w_/M_n_ = 1.61 by GPC). The allyl-terminated polyisobutylene (PIB-All) used in this work was obtained by quasiliving carbocationic polymerization of isobutylene and in-situ quenching by ATMS [32]. This polymer has 100% allyl functionality according to the ^1^H NMR spectrum of this polymer (Figure 1 and Figure S2), and M_n_ of 820 g/mol and M_w_/M_n_ of 1.16. The reaction routes for obtaining hydroxyl-ended PIBs from these olefin-terminated polymers are depicted in Scheme 1. As expected, hydroboration/oxidation with 9-BBN [31] converts only the exo-olefin in PIB-Exo to hydroxyl groups as indicated in Figure 2 and Figure S4. The endo-olefin and in-chain double bonds do not react due to steric hindrance. The allyl endgroup is fully transformed to hydroxyl group as shown in Figure 3 and Figure S3. The resulting hydroxyl-ended PIBs, that is PIB_exo_-OH and PIB_all_-OH, react with tosyl chloride (TsCl) and nosyl chloride (NsCl) as displayed in Scheme 1 under various reaction conditions. All reactions were carried out under dry atmosphere at room temperature.

The first attempts on tosylation of PIB_exo_-OH were carried out to reproduce previously described experiments [47] by applying the same reaction conditions (DCM solvent, 2 eq. of TsCl, 2 eq. of DMAP, 13.7 eq. of TEA, room temperature, 10 h reaction time). However, surprisingly these conditions did not lead to full conversion of the PIB_exo_-OH. Therefore we tried also pyridine instead of DMAP, different TsCl concentrations and various reactions times (Entry 1–3 in Table 1). As shown in Table 1, pyridine alone is not sufficiently effective catalyst of tosylation, leading only to 16% conversion. As the data indicate in Table 1 and in Figure 2, tenfold excess of TsCl in the presence of 2 eq. DMAP and 10 eq. TEA leads to complete transformation of the hydroxyl groups in PIB_exo_-OH to PIB with tosyl endgroups (PIB_exo_-OTs), even with relatively short reaction time of 7 h (Entry 5 in Table 1). This reaction time is sufficient to reach only 80% conversion during nosylation under the otherwise same condition, but using THF as solvent, as shown in Table 2 (Entry 11). Because the nosyl chloride has limited solubility in DCM, all nosylation reactions were carried out in THF, which is an appropriate solvent for both NsCl and PIB. Longer reaction times of nosylation of PIB_exo_-OH lead to ~90% conversion, i.e., to the formation of nosyl ended polymers (PIB_exo_-ONs) as indicated by the ^1^H NMR spectrum in Figure 2.

**Scheme 2:**
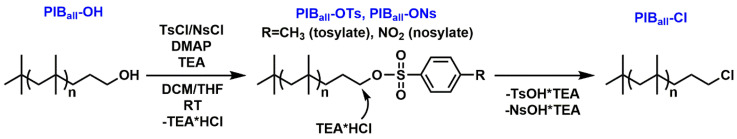
Tosylation/nosylation of hydroxyl-ended polyisobutylene (PIB_all_-OH) and subsequent chlorination leading to primary chloride endfunctional polyisobutylene.

**Figure 4:**
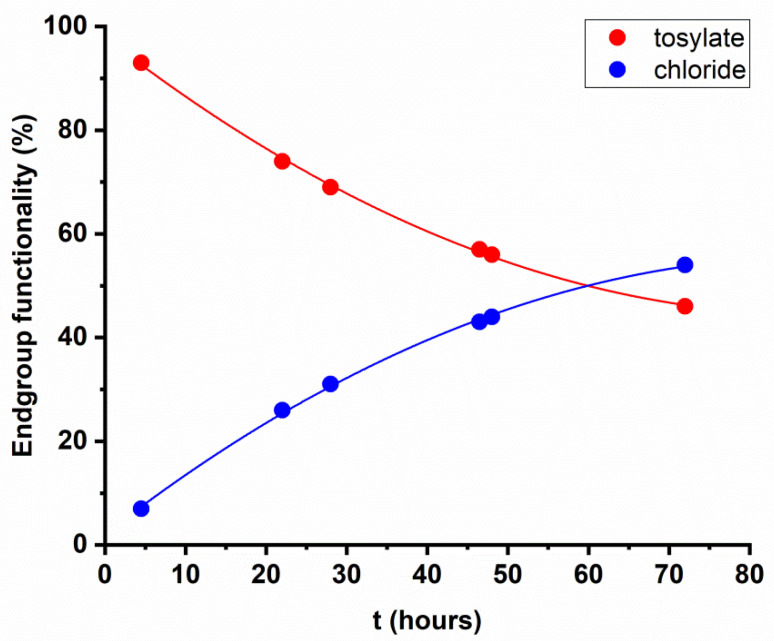
Tosylate and chloride endgroup functionalities as a function of the reaction time of the tosylation of PIB_all_-OH (Entry 19–24 in Table 3; the lines are drawn only for directing the eye).

The results of the experiments on the sulfonylation of PIB_exo_-OH indicated that 10 eq. of the alkyl sulfonyl chloride with the combination of 2 eq. DMAP and 10 eq. TEA results in complete transformation of the hydroxyl group for tosylation and nearly complete conversion for nosylation. Because the PIB-Exo has an extra methyl group connected to the chain terminus compared to the lack of such substituent in the allyl endgroup of PIB-All, it was expected that the latter one has higher reactivity than PIB_exo_-OH. However, using smaller excess of tosyl chloride, that is, only 2–5 eq., for the tosylation of PIB_all_-OH resulted in only partial conversion even with longer reaction times as shown in Table 3 (Entry 15–18), on the one hand. On the other hand, alkyl chloride chain end appeared at long reaction times (72 h). Thus, experiments with tenfold excess of TsCl reagent were carried out by using the same reaction conditions as for PIB_exo_-OH with reaction times from 4.5 h to 72 h (Entry 19–24 in Table 3). These experiments led to interesting results. First, the transformation of the alcohol chain end is much faster with PIB_all_-OH, i.e., the reactivity of PIB_all_-OH is higher than that of PIB_exo_-OH with TsCl as expected. Second, not only tosyl endgroups but PIBs bearing a terminal primary chlorine group (PIB_all_-Cl) as side products were also observed as indicated by the signal at 3.50 ppm in the ^1^H NMR spectra which is assigned to the methylene group next to the chlorine termini (Figure 3). Furthermore, the amount of PIB_all_-Cl is increasing, while the amount of tosylated PIB is decreasing with the reaction time as shown in Table 3 (Entry 19–24) and in Figure 4. Therefore, the occurrence of the following processes can be considered to take place according to results with low MW compounds [39] under the applied conditions as depicted in Scheme 2: (1) the initial reaction of the alkyl sulfonyl chloride with the alcohol yields the main tosylated product, (2) meanwhile the HCl byproduct forms triethylammonium hydrochloride (TEA*HCl) with TEA, in which the chloride ion has a sufficient nucleophilicity to displace the alkyl sulfonyl group. As a consequence, as can be seen in Scheme 2, the first substitution step, i.e., tosylation, is followed by substituting the tosyl group, which is a good leaving group, with chlorine.

Experiments with lower than 10 eq. TEA were also carried out. It was found that although 2 eq. TEA results in 5% chloride endgroup (Entry 25), which is much smaller than that obtained with 10 eq. (Entry 20 in Table 3) at similar reaction time. However, only 53% of the hydroxyl group is consumed in this reaction (Entry 25 in Table 3). Increasing the amount of TEA to 3 eq. and 5 eq. leads to complete conversion of the hydroxyl groups and nearly 90% tosylate functionality is observed with 5 eq. of TEA in 20 h reaction time. Summarizing the results of tosylation of PIB_all_-OH with TsCl obtained with DMAP catalyst in the presence of TEA in the range of 5–10 eq. it can be claimed that with 10 eq. TsCl PIB-tosylates with around 90% tosyl functionalities and ~10% of PIB_all_-Cl can be prepared. As reported in the literature [40], not only DMAP but 1-methylimidazole (1-MI) is also an efficient catalyst of acylation of alcohols. Therefore, attempts were made by us to investigate the effect of 1-MI on the tosylation reaction of PIB_all_-OH by still keeping the amount of TEA at relatively low levels as shown in Table 3. As indicated by the results of Entry 30–34, 2 eq. TEA is insufficient to reach high tosylation yields, but with 3 eq. and 5 eq. of TEA, the consumption of the hydroxyl groups is complete, the undesired side reaction of chlorination is suppressed to 3–8%, and thus PIB_all_-tosylates with 92–97% tosylate endfunctionalities are obtained as confirmed by the ^1^H NMR spectrum in Figure 3. These optimal reaction conditions by using 1-MI as catalyst of tosylation of PIB_all_-OH provide an efficient tool to prepare tosylate-ended PIBs with high, nearly quantitative endfunctionalities. Due to the fact that the PIB-All prepolymer with narrow MWD is synthesized by quasiliving carbocationic polymerization of isobutylene, well-defined PIB_all_-OTs also with narrow MWD can be obtained by this process. Thus, the resulting PIB_all_-OTs can be utilized in various further derivatization reactions and as a starting material for macromolecular assemblies of complex architectures.

The effect of the reaction conditions on the nosylation of PIB_all_-OH was also investigated. As shown in Table 4, the consumption of the terminal hydroxyl groups is complete in the presence of 10 eq. NsCl, 2 eq. DMAP and 10 eq. TEA after 4 h reaction time (Entry 35–39). However, with this and longer reaction times from 14% to 30% chlorine endgroups are also present in the resulting polymers. Decreasing the amount of TEA to 2 eq. gives only 14% nosylation conversion in 19 h. Using reduced amounts of TEA of 3 eq. and 5 eq. results in complete hydroxyl consumption, and in the case of 5 eq. TEA 91% and 93% nosyl endfunctionalities are observed (Entry 45 and 46 in Table 4). With 1-MI as catalyst, PIB_all_-ONs with 89–94% nosyl endfunctionalities are formed as the ^1^H NMR spectra and the data in Table 4 indicate (Figure 3). In these cases, negligible amounts of 2–5% PIB_all_-ONs with unreacted hydroxyl termini can also be observed, and the chlorine endfunctionalities fall in the region of 4–8%. These results show that PIB_all_-ONs with higher than 90% nosyl functionalities can be obtained with either DMAP or 1-MI catalyst by using 5 eq. of TEA and proper reaction times. Based on the reactivity of the nosyl group, these novel nosyl-ended PIBs, unpublished so far in the open literature according to the best of our knowledge, are expected to open new ways for the preparation of various PIB-based polymer architectures.

## 4. Conclusions

Tosylation and nosylation of hydroxyl-ended polyisobutylenes (PIB-OHs) derived from a commercially available exo-olefin-terminated polymer (PIB_exo_-OH) and from allyl-ended macromolecules (PIB_all_-OH), prepared by quasiliving carbocationic polymerization of isobutylene, were systematically investigated. A thorough exploration was conducted to reveal the influence of the ratios of the reagents, such as 4-dimethylaminopyridine (DMAP), pyridine, 1-methylimidazole (1-MI), and trimethylamine (TEA), and reaction time on the conversion of the hydroxyl termini in these PIB-OHs. A significant difference in the reactivity between the two hydroxyl-terminated polymers was found, i.e., the PIB_all_-OH reacts faster with the sulfonyl chlorides than the PIB_exo_-OH, presumably because of steric reasons. While quantitative tosylation was achieved with PIB_exo_-OH, nosylation led to PIB_exo_-ONs with functionality of ~90%. Unexpectedly, it was found that the tosyl endgroup reacts further with the chloride ion formed during tosylation, and chlorine-ended PIB (PIB_all_-Cl) is formed. The conversion of the hydroxyl group and the relative amount of the sulfonyl and chlorine termini strongly depend on TEA and reaction times. Decreased amounts of TEA in the range of 3–5 eq. and optimal reaction times lead to PIB_all_-OTs and PIB_all_-ONs with higher than 90% sulfonyl functionalities. The resulting tosyl- and nosyl-ended PIBs are capable of subsequent derivatizations, and thus various novel endfunctional PIBs can be obtained via substitution reactions. This enables the preparation of an array of PIB-containing new macromolecular architectures not existed before.

## Figures and Tables

**Table 1:** The effect of the reaction conditions on the tosylation of PIB_exo_-OH at room temperature.

**Entry**	**Solvent**	**t (h)**	**TsCl (eq.)**	**Catalyst (eq.)**	**TEA (eq.)**	**-OH (%)**	**-OTs (%)**
**1**	DCM	24	1.25	4 (pyridine)	0	84	16
**2**	DCM	48	1.25	4 (pyridine)	0	84	16
**3**	DCM	72	1.25	4 (pyridine)	0	84	16
**4**	DCM	24	5	2 (DMAP)	10	25	75
**5**	DCM	7	10	2 (DMAP)	10	0	100
**6**	DCM	22	10	2 (DMAP)	10	0	100
**7**	DCM	24	10	2 (DMAP)	10	0	100
**8**	DCM	29	10	2 (DMAP)	10	0	100
**9**	DCM	48	10	2 (DMAP)	10	0	100
**10**	DCM	70	10	2 (DMAP)	10	0	100

**Table 2:** The effect of the reaction conditions on the nosylation of PIB_exo_-OH at room temperature.

**Entry**	**Solvent**	**t (h)**	**NsCl (eq.)**	**Catalyst (eq.)**	**TEA (eq.)**	**-OH (%)**	**-ONs (%)**
**11**	THF	7	10	2 (DMAP)	10	20	80
**12**	THF	22	10	2 (DMAP)	10	11	89
**13**	THF	29	10	2 (DMAP)	10	12	88
**14**	THF	70	10	2 (DMAP)	10	10	90

**Table 3:** The effect of the reaction conditions on the tosylation of PIB_all_-OH at room temperature.

Entry	Solvent	t (h)	TsCl (eq.)	Catalyst (eq.)	TEA (eq.)	–OH (%)	–OTs (%)	–Cl (%)
15	DCM	24	2	2 (DMAP)	10	83	17	0
16	DCM	72	3	2 (DMAP)	10	18	61	21
17	DCM	24	5	2 (DMAP)	10	33	67	0
18	DCM	72	5	2 (DMAP)	10	51	29	20
19	DCM	4.5	10	2 (DMAP)	10	0	93	7
20	DCM	22	10	2 (DMAP)	10	0	74	26
21	DCM	28	10	2 (DMAP)	10	0	69	31
22	DCM	46.5	10	2 (DMAP)	10	0	57	43
23	DCM	48	10	2 (DMAP)	10	0	56	44
24	DCM	72	10	2 (DMAP)	10	0	46	54
25	DCM	19	10	2 (DMAP)	2	47	48	5
26	DCM	20	10	2 (DMAP)	5	0	89	11
27	DCM	50	10	2 (DMAP)	5	0	81	19
28	DCM	20	10	3 (DMAP)	3	0	84	16
29	DCM	50	10	3 (DMAP)	3	0	79	21
30	DCM	19	10	2 (1-MI)	2	40	58	2
31	DCM	20	10	2 (1-MI)	5	0	97	3
32	DCM	50	10	2 (1-MI)	5	0	94	6
33	DCM	20	10	3 (1-MI)	3	0	97	3
34	DCM	50	10	3 (1-MI)	3	0	92	8

**Table 4:** The effect of the reaction conditions on the nosylation of PIB_all_-OH at room temperature.

9	Solvent	t (h)	NsCl (eq.)	Catalyst (eq.)	TEA (eq.)	-OH (%)	-ONs (%)	-Cl (%)
35	THF	1	10	2 (DMAP)	10	8	89	3
36	THF	2	10	2 (DMAP)	10	3	93	4
37	THF	3	10	2 (DMAP)	10	2	94	4
38	THF	4	10	2 (DMAP)	10	2	92	6
39	THF	4.5	10	2 (DMAP)	10	0	86	14
40	THF	22	10	2 (DMAP)	10	0	82	18
41	THF	28	10	2 (DMAP)	10	0	82	18
42	THF	46.5	10	2 (DMAP)	10	0	81	19
43	THF	72	10	2 (DMAP)	10	0	71	29
44	THF	19	10	2 (DMAP)	2	86	9	5
45	THF	20	10	2 (DMAP)	5	1	92	7
46	THF	50	10	2 (DMAP)	5	0	91	9
47	THF	20	10	3 (DMAP)	3	0	81	19
48	THF	50	10	3 (DMAP)	3	0	72	28
49	THF	19	10	2 (1-MI)	2	49	38	13
50	THF	20	10	2 (1-MI)	5	2	94	4
51	THF	50	10	2 (1-MI)	5	2	90	8
52	THF	20	10	3 (1-MI)	3	5	91	4
53	THF	50	10	3 (1-MI)	3	5	89	6

## Supplementary Materials

The following are available online at https://www.mdpi.com/2073-4360/12/11/2504/s1, Figure S1: 1H NMR spectrum of the PIB-Exo sample with integral values; Figure S2: 1H NMR spectrum of the PIB-All sample with integral values; Figure S3: 1H NMR spectrum of the PIBall-OH sample with integral values; Figure S4: 1H NMR spectrum of the PIBexo-OH sample with integral values; Figure S5: 1H NMR spectrum of the PIBall-OTs sample with integral values; Figure S6: 1H NMR spectrum of the PIBall-ONs sample with integral values; Figure S7: GPC curves of the synthesized PIBall-OTs macroinitiator and its starting material PIBall-OH; Figure S8: GPC curves of the synthesized PIBall-ONs macroinitiator and its starting material PIBall-OH.
