# Supplementary material for: Synthesis of Tosyl- and Nosyl-Ended Polyisobutylenes with High Extent of Functionalities: The Effect of Reaction Conditions
Source: Polymers (Basel). 2020 Oct 28;12(11):2504. doi: 10.3390/polym12112504 (PMC7692378; doi:10.3390/polym12112504)
Supplement: Supplementary file 1 [file polymers-12-02504-s001.pdf]

Supporting Information

Synthesis of Tosyl- and Nosyl-ended Polyisobutylenes with High Extent of Functionalities: The Effect of Reaction Conditions

Balázs Pásztói[1,2]*, Tobias M. Trötschler[3,4,5], Ákos Szabó[1], Györgyi Szarka[1], Benjamin Kerscher[3,4], Rolf Mülhaupt[3,4,5]*, Béla Iván[1]*

[1]Polymer Chemistry Research Group, Institute of Materials and Environment Chemistry, Research Centre for Natural Sciences, Magyar tudósok körútja 2, Budapest, H-1117, Hungary
[2]George Hevesy PhD School of Chemistry, Institute of Chemistry, Faculty of Science, Eötvös Loránd University, Pázmány Péter sétány 2, H-1117 Budapest, Hungary
[3]Institute for Macromolecular Chemistry, University of Freiburg, Stefan-Meier-Str. 31, D-79104 Freiburg, Germany
[4]Freiburg Materials Research Center, University of Freiburg, Stefan-Meier-Str. 21, D-79104 Freiburg, Germany
[5]Freiburg Center for Interactive Materials and Bioinspired Technologies (FIT), University of Freiburg, Georges-Köhler-Allee 105, D-79110 Freiburg, Germany

***** Correspondence: pasztoi.balazs@ttk.hu (B. P.), rolf.muelhaupt@makro.uni-freiburg.de (R. M.), ivan.bela@ttk.hu (B. I.)

1. ^{1}H NMR spectra

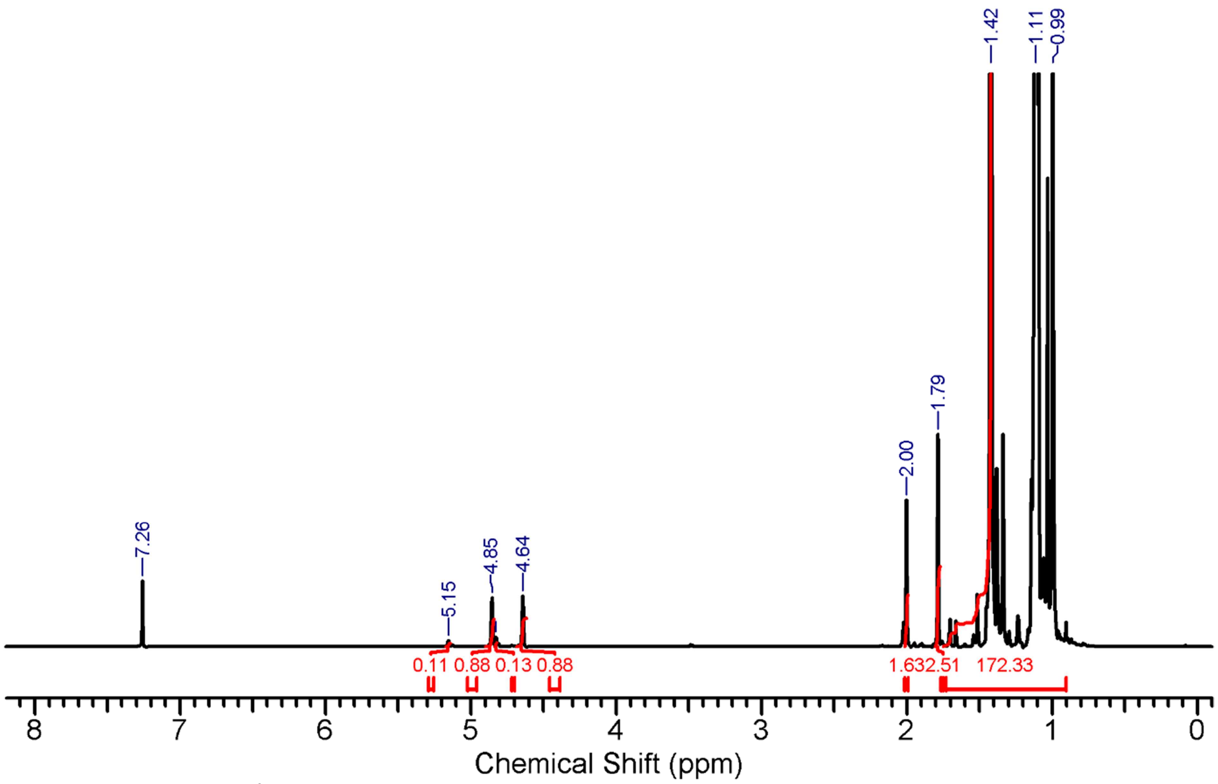

Figure S1. ^{1}H NMR spectrum of the PIB-Exo sample with integral values.

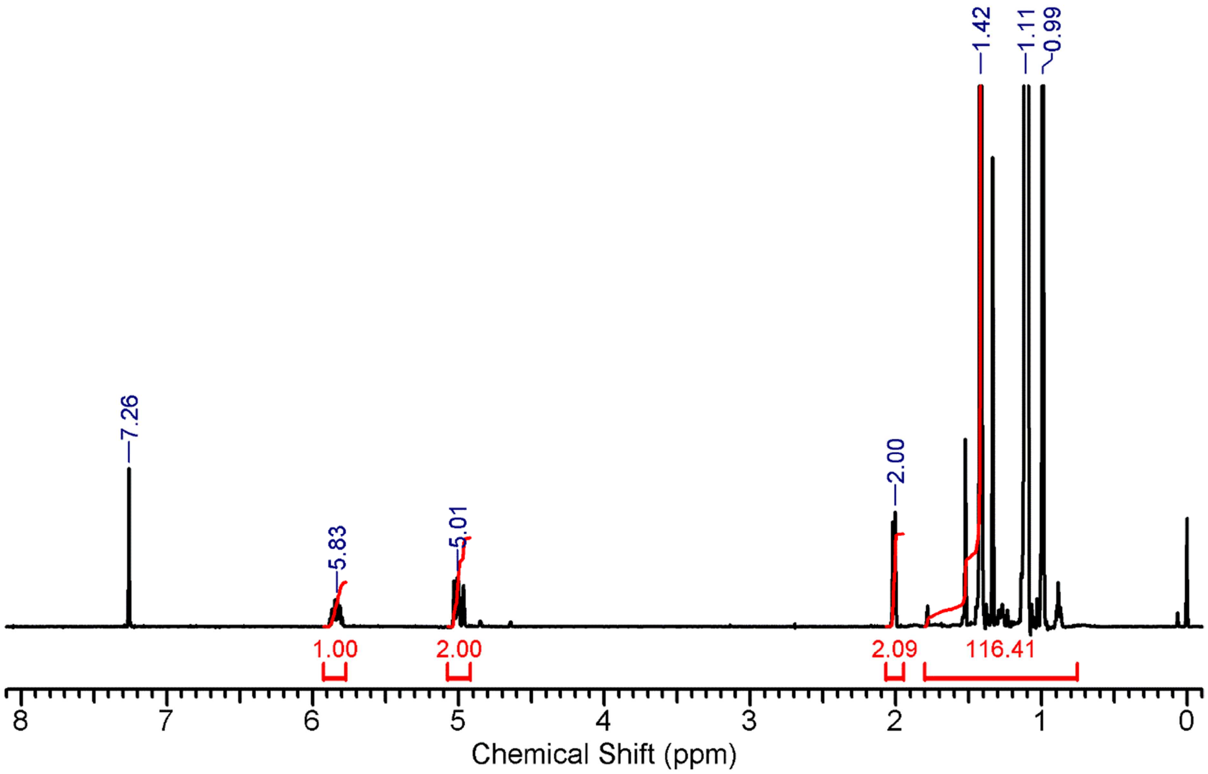

Figure S2. ^{1}H NMR spectrum of the PIB-All sample with integral values.

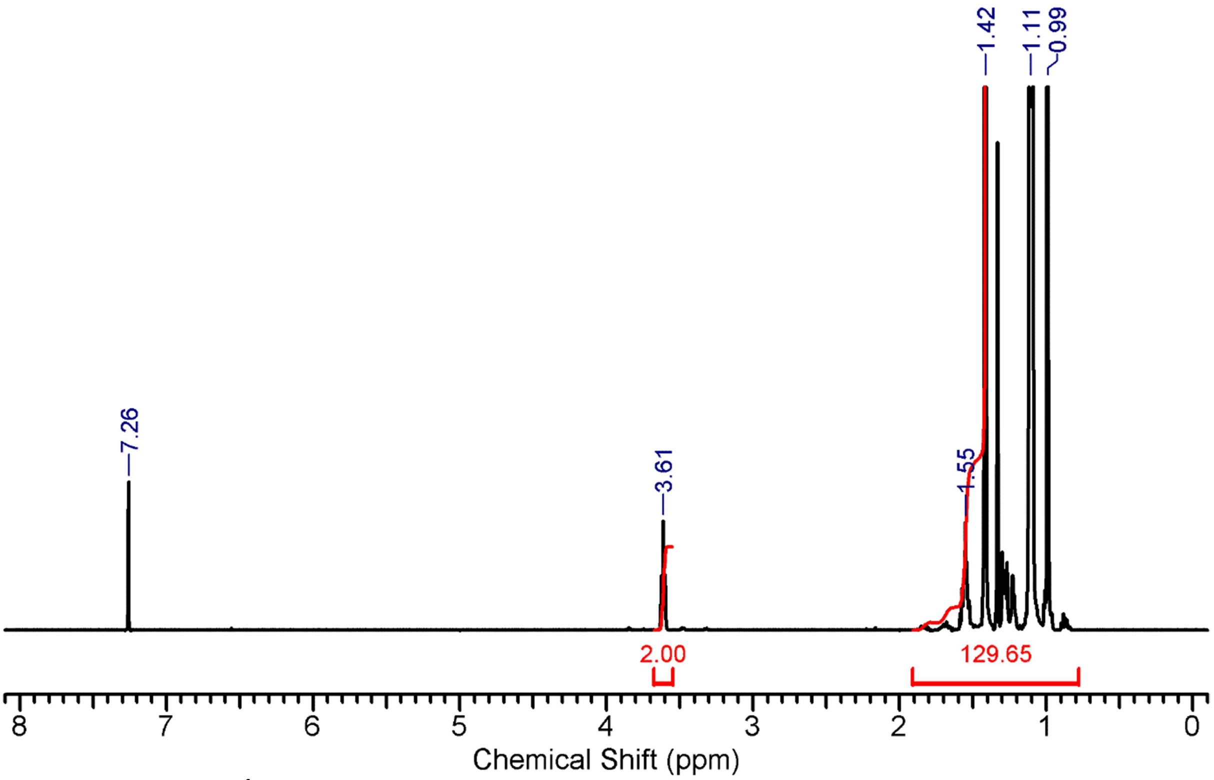

Figure S3. ^{1}H NMR spectrum of the PIB_{all}-OH sample with integral values.

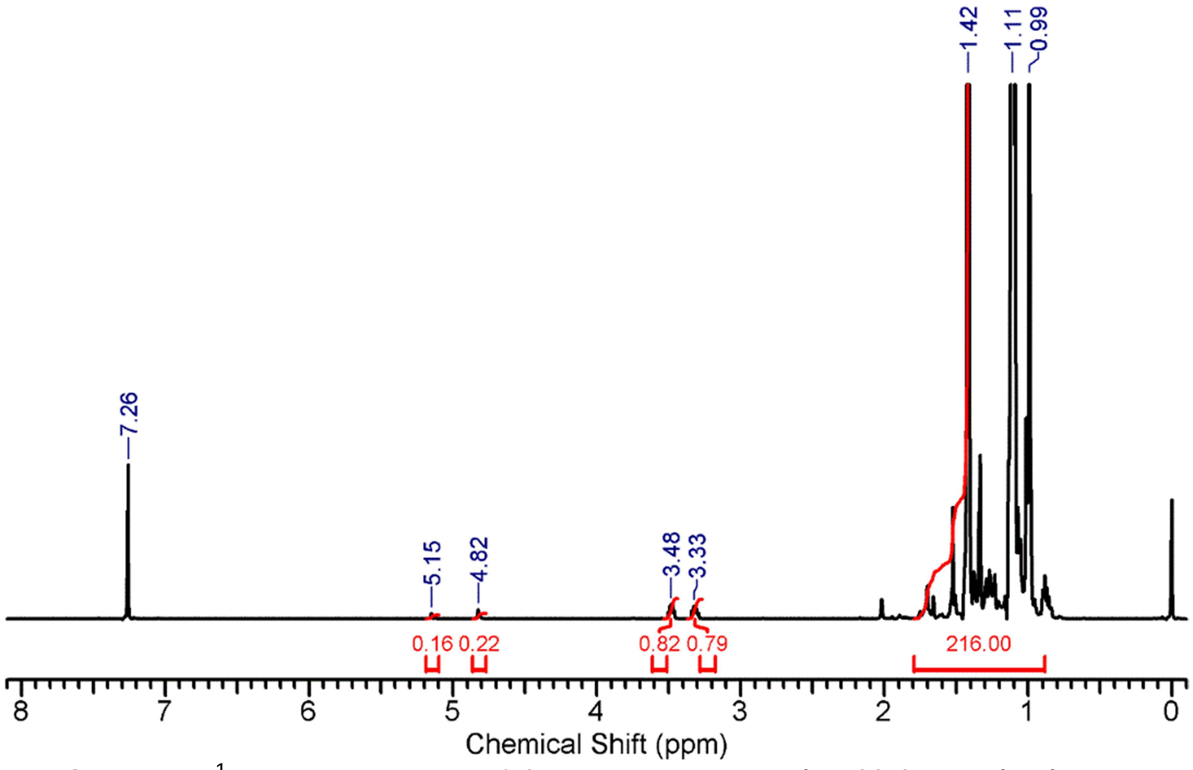

Figure S4. ^{1}H NMR spectrum of the PIB_{exo}-OH sample with integral values.

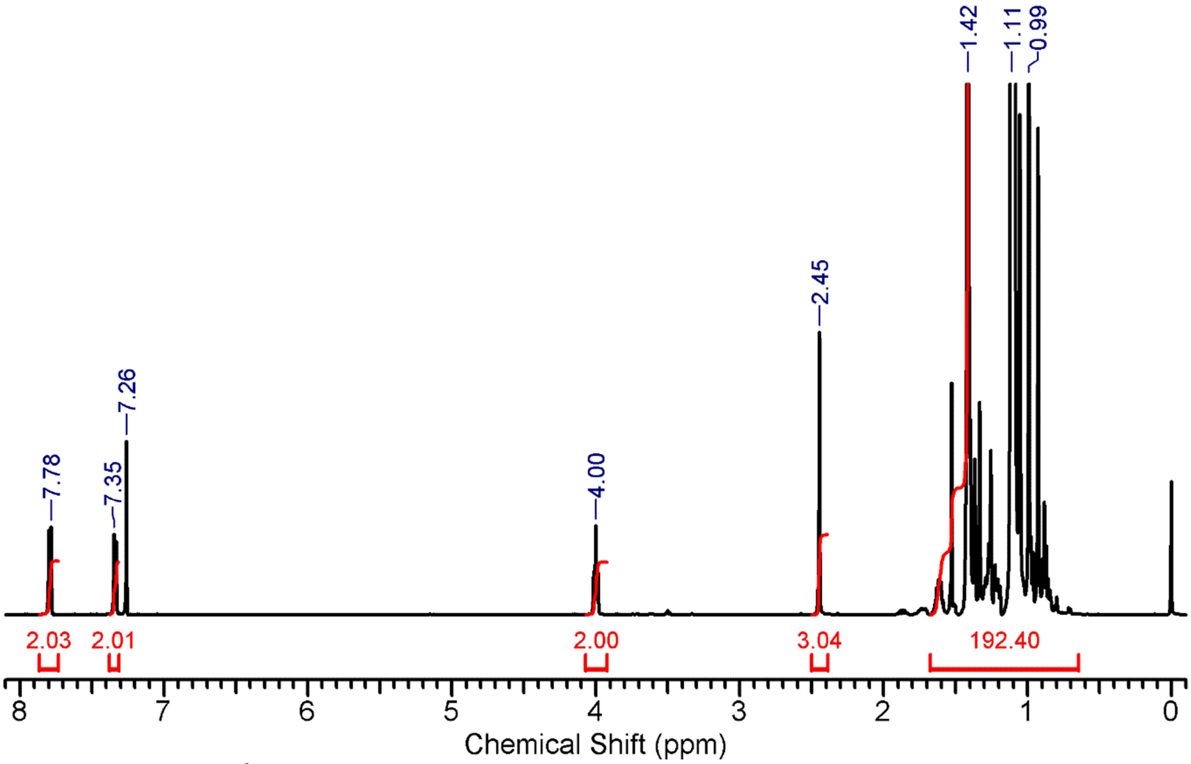

Figure S5. ^{1}H NMR spectrum of PIB_{all}-OTs sample with integral values.

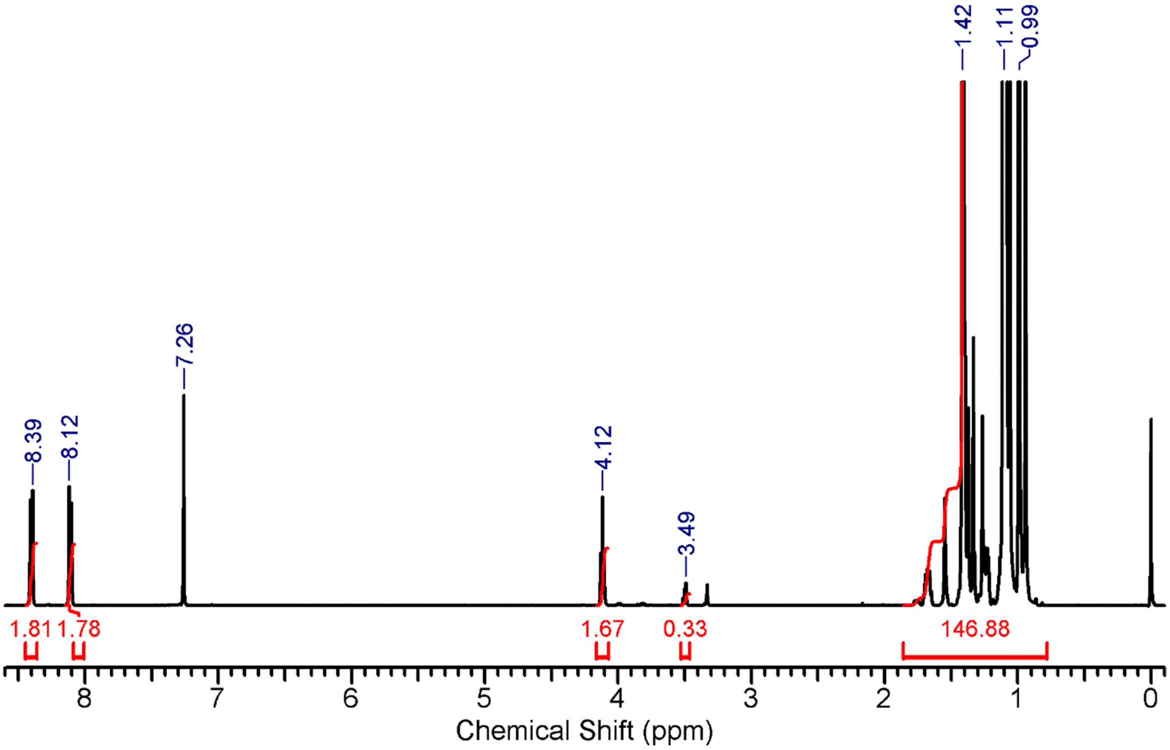

Figure S6. ^{1}H NMR spectrum of the PIB_{all}-ONs with integral values.

2. GPC curves

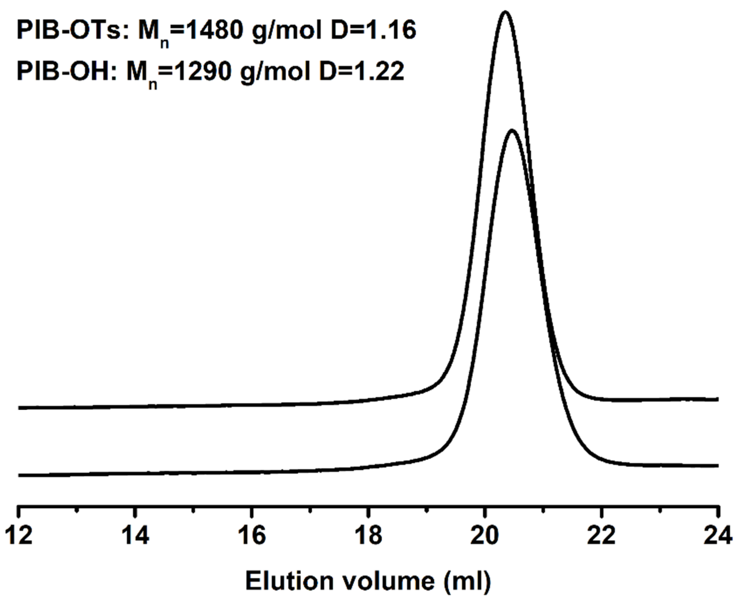

Figure S7. GPC curves of the synthesized PIB_{all}-OTs macroinitiator and its starting material PIB_{all}-OH.

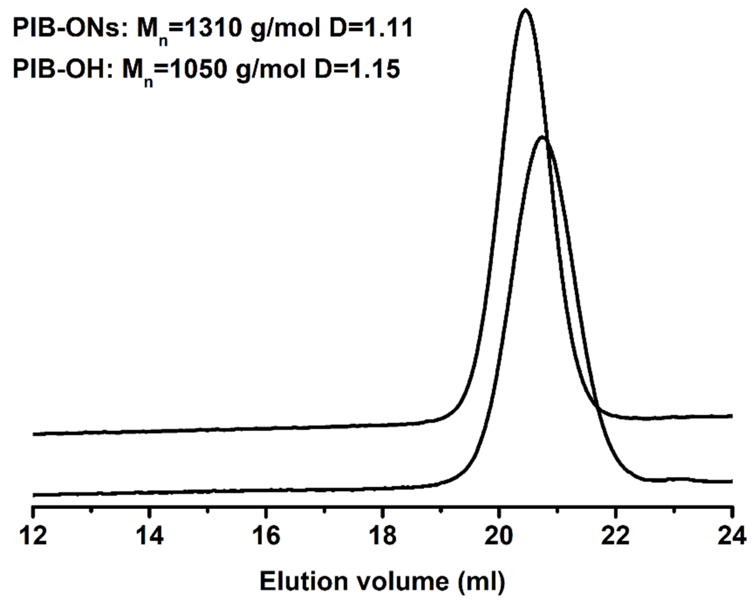

Figure S8. GPC curves of the synthesized PIB_{all}-ONs macroinitiator and its starting material PIB_{all}-OH.
